# 肺部肿瘤的精确放射治疗研究进展

**DOI:** 10.3779/j.issn.1009-3419.2011.11.12

**Published:** 2011-11-20

**Authors:** 峰 许

**Affiliations:** 1 610041 成都，四川大学华西医院肿瘤中心放疗科 Department of Radiation Oncology, Cancer Center, West China Hospital, Sichuan University, Chengdu 610041, China; 2 6140041 成都，四川大学华西医院肿瘤中心国家生物治疗重点实验室 State Key Laboratory of Biotherapy, Cancer Center, West China Hospital, Sichuan University, Chengdu 610041, China

**Keywords:** 肺肿瘤, 放射治疗, 治疗, Lung neoplasms, Radiotherapy

## Abstract

目前肺部肿瘤的放射治疗已进入精确放疗时代。实施精确放疗的具体方法主要包括：调强放疗（intensity modulated radiotherapy, IMRT）、图像引导放射治疗（image-guided radiotherapy, IGRT）和体部立体定向放射治疗（stereotactic body radiotherapy, SBRT）。在实施精确放疗过程中，对于以下问题：患者体位固定、肺部肿瘤运动的控制、影像技术的使用、PTV边界、剂量的处方和报道、射野的安排、剂量体积的控制和治疗的实施等，应给予充分的考虑和注意，以确保精确放疗能够精确执行。

## Abstract (en)

At present lung tumor radiation therapy has entered the accurate radiotherapy era. Precise radiotherapy includes intensity modulated radiotherapy (IMRT), image-guided radiotherapy (IGRT) and stereotactic body radiotherapy (SBRT). During the process of implementing precise radiotherapy, these problems should be fully considered to ensure executing precise radiotherapy accurately: patient positioning, controlling of the lung tumor motion, selecting of image techniques, PTV margin, dose prescription and reporting, arrangement of beams, controlling of dose volume and treatment delivering.

放射治疗是肺部恶性肿瘤的重要治疗方法之一。但由于肺部肿瘤受呼吸运动的影响而存在一定程度的位移; 并且其周围的危险器官（organs at risk, OARs），尤其是正常肺组织的放疗耐受性较差等问题，给放射治疗带来了很大的挑战。但是随着现代放射治疗技术的飞速发展，放疗技术已经从使用简单的方形野的二维照射发展到了今天的适形度和精确度越来越高的三维适形放疗（three dimensional conformal radiotherapy, 3D-CRT）、调强放疗（intensity modulated radiotherapy, IMRT）、图像引导放射治疗（image-guided radiotherapy, IGRT）等。这种转变带来的结果是在减少周围危险器官受照剂量的同时提高了肿瘤区的放射剂量，从而进一步提高了治疗效果。此外对于肿瘤靶区的运动等问题目前也有了较为清晰的认识，并得到了较为理想的解决，这就更进一步保证了精确放疗计划能够得以精确实施。

目前肺部肿瘤的放射治疗已经进入精确放疗时代。为了更好地认识和理解肺部肿瘤的精确放疗，现将实施精确放疗的具体方法和实施过程中应特别注意的问题综述如下。

## 1 实施精确放疗的具体方法

### 1.1 IMRT

IMRT是在3D-CRT的基础上发展而来的，其特点是可以在治疗区域内形成凹陷的、不规则的、高度适合靶区形状的剂量分布，从而达到剂量绘画和剂量雕刻的效果。

IMRT的实施可以使用静态或动态多叶光栅（multi-leaf collimators, MLC）。前者即分步照射IMRT（step and shoot IMRT），后者包括断层治疗（tomotherapy）和容积旋转调强放疗（volumetric arc modulated therapy, VMAT）等。这3种类型的放疗方法各有优缺点。例如静态MLC更加简单且易于实现，但效率较低、存在子野位置验证等问题; 断层治疗需要特殊的治疗设备才能实施，存在窄野的匹接及对床步进的精确度要求甚高等问题; VMAT具有使用整野治疗、不必将野分成窄束、不存在相邻窄野间的匹接、治疗实施的时间明显减少等优势，但因其要求叶片运动必须与输出强度调制同步等，故对加速器的控制精度等要求颇高，还需在技术上进一步完善。

此外，同步加量放疗也是IMRT的一大优势，即可以在同一时期内实现不同靶区接受不同剂量照射的目的。这就避免了射野衔接和补充电子线的问题，从而可以降低剂量的不确定性。

对正常组织的保护方面，IMRT已经具有了相当的优势。在此基础上，若想进一步提高IMRT的治疗比，可以采用以下方法^[1]^，如：①利用调强质子放疗（intensity modulated proton therapy, IMPT）技术降低正常组织结构的剂量; ②联合正电子发射计算机断层显像（positron emission tomography, PET）等确定生物靶区，以利于更进一步选择性提高生物靶区的剂量; ③通过调整分割剂量来提高肿瘤的剂量效应，如使用立体定向放射外科（stereotactic radiosurgery, SRS）技术等; ④联合使用影像引导装置使治疗得到更好验证; ⑤开发更精确的软件进行在线或离线调整以减小治疗实施过程中的误差。

总体而言，IMRT的主要应用病种是头颈部肿瘤和前列腺癌，但近年来IMRT在肺部肿瘤中的应用也得到了很大的发展^[2-4]^。

### 1.2 IGRT

有研究^[5]^结果显示，通过容积影像所测量得的摆位误差、器官运动、肿瘤及正常组织的外形及体积的变化等，在某些情况下会超乎想象，但图像引导的使用可以降低其对治疗造成的负面影响^[6-8]^。图像引导是一种可以检测出治疗过程中系统误差和随机误差的有效工具，针对检测出的误差，可采取相应的措施予以应对，从而有效地达到缩小靶区边界的作用。射野影像（portal imaging）和数字化重建影像（digitally reconstructed radiographs, DRRs）是图像引导的基本形式。而目前正在应用的更加先进的IGRT技术可以通过相应的影像技术直接确定靶区的位置，而不需要通过患者体位来进行间接的推断。影像引导可以提高靶区定位的准确性，并可以调整治疗过程中的分次间和分次内的靶区运动。对于摆位误差和分次间的位移，可以采用在线调整或自适应放疗技术; 对于分次内的位移，可采用呼吸控制技术和四维放疗技术，或实时跟踪技术。

总体而言，现阶段IGRT的作用主要体现在以下几个方面：

#### 1.2.1 缩小靶区边界

摆位误差和器官运动的存在会造成肿瘤区域的漏照或OARs的过量照射。针对这一问题，最常用的处理方法是在临床靶区（clinical target volume, CTV）的基础上外放一定的边界以形成计划靶区（plan target volume, PTV）。该边界又可分为两部分：考虑到器官的生理变化及运动等因素的基础上外放的内边界（internal margin, IM）以及考虑到摆位误差及治疗机照射野位置的变化等因素情况下外放的摆位边界（set-up margin, SM）。即PTV=CTV+IM+SM，其中CTV + IM又被称作内靶区（internal target volume, ITV）^[9]^。通过减小呼吸动度、采用呼吸门控技术以及采用实时跟踪技术等可以明显缩小IM。而SM大小的确定依赖于以下因素：体位固定装置的类型、确定和调整摆位误差的方法，如是否使用锥形束CT（cone beam CT, CBCT）等^[10, 11]^。通过IGRT的在线调整能够明显缩小SM。而靶区边界的缩小则有助于同时降低OARs的受照剂量并提高靶区的照射剂量。此外，还可以通过调整剂量分布、改变剂量分割方式以及提高照射剂量来达到提高治疗比的目的^[1]^。

#### 1.2.2 自适应性放疗（adaptive radiotherapy, ART）

PTV-CTV的间距是根据患者的群体摆位误差和器官运动数据设定的。但具体到每一位患者而言，其实际所需要的间距是不同的，应该具有一定的个体差异。因此有必要考虑使用个体化边界^[12]^。ART正是为了这一目的而设计的。其过程是，自疗程开始每次治疗时均获取患者的二维或三维图像，用离线方式测量每次治疗前的摆位误差; 根据最初数次（5次-9次）的测量结果来预测整个疗程的摆位误差，然后根据该结果来调整PTV和CTV的间距，进而修改治疗计划，再按修改后的计划实施后续治疗。除此而外，还可以进一步根据患者实际照射过程中的受照剂量或肿瘤对治疗的相应变化等反馈信息，对治疗方案做相应的调整。

#### 1.2.3 治疗准确性的验证

IGRT另一十分重要的作用是治疗准确性的验证。验证的实施不仅在治疗前，通常还贯穿于治疗的整个过程; 这可以将系统误差和随机误差减小从而避免肿瘤区的欠量和OARs的超量。

### 1.3 体部立体定向放射治疗（stereotactic body radiotherapy, SBRT）

近年来，SBRT的研究和应用得到了飞速的发展，并越来越成为国际放射肿瘤界的热点。SBRT是将多源、多线束或多野高能射线在三维空间上聚焦于体内某一靶区，形成一个围绕焦点的高剂量区进行照射，并且其剂量强度从焦点中心向边缘逐渐衰减，到达周围正常组织后剂量下降十分陡峭，从而更有利于靶区剂量的提升和周围正常组织的保护。其主要优势在于分次剂量高、分割次数少、总疗程短、靶区受照剂量高而正常组织受照剂量较低。由于SBRT采用的大分割放疗可以降低肿瘤细胞的再增殖，因此可以获得比常规分割放疗更好的疗效。

SBRT可以通过直线加速器或赛博刀（cyberknife, Accuray Inc, CA, USA）加以实施。这种技术目前越来越多地被应用于Ⅰ期肺癌的根治性放疗以及肾细胞癌、肝细胞癌、椎体肿瘤和前列腺癌（首程治疗或术后补量照射）的放疗等。目前SBRT在转移性肺癌和肝癌的治疗中也显示出明显的优势^[13]^。

由此可见，目前实现精确放射治疗的手段和方法是多种多样的。在选择治疗方案时，应该根据患者的具体情况进行恰当的选择。

## 2 实施肺部肿瘤精确放疗过程中的注意事项

精确放射治疗主要体现为精确定位、精确计划和精确治疗这三个方面的精度控制。这除了要求减少系统误差，还要求减少各个阶段的随机误差。下面针对可以提高放疗精度的多个环节及相应的处理方法进行综述。

### 2.1 患者体位的固定

患者体位的固定性和重复性十分重要，这也是实现精确定位的前提条件之一。如果有可能，患者摆位时尽量采用双手上举的体位，这种摆位方式有利于射野方向的任意选择以达到更好的靶区覆盖并保护正常组织。尽管有研究^[14]^报道SBRT可以在没有体位固定装置时安全实施，但欧洲癌症治疗研究组织（European Organisation for Research and Treatment of Cancer, EORTC）仍然推荐对SBRT的患者进行更高精度的体位固定^[15]^。

### 2.2 肺部肿瘤运动的控制

如前所述，运动是肺部肿瘤放射治疗过程中的一个重大挑战，尤其是对于目前实施的精确放射治疗而言至关重要。通过透视和四维CT观测到的肺下叶肿瘤的运动范围可达到9.1 mm-31.22 mm^[16-19]^。由此可见，肺部肿瘤的运动会导致治疗时靶区的位移以及放疗剂量的不精确^[20]^。

要达到限制器官运动度、减小PTV边界的目的，可以采取多种方法^[1, 21]^：①压腹^[22, 23]^，该技术可以减少肿瘤的运动幅度，但会增加肿瘤运动幅度的可变性^[24, 25]^;②呼吸门控技术^[16, 26-28]^，包括一个内置的参考标记和一个外在的呼吸信号检测设备。目前该技术仍处于研究阶段^[15]^;③靶区跟踪技术^[29, 30]^，该技术仅适用于小部分肿瘤位移明显且能够实施以肿瘤为基础的在线调整的患者^[31]^。该技术的使用尚需要进一步前瞻性成本效应的研究进行评价; ④呼吸控制技术，可分为主动和被动呼吸控制^[20, 32-35]^。其中主动呼吸控制技术（active breath control, ABC）是一种相对简单且有效的方法^[32, 35]^，但不是所有患者均能耐受; 且外在的参照如呼吸状态或胸壁、膈肌的位移等并不能充分反映肺内肿瘤或内置标记物的确切位置变化^[36]^。

关于呼吸控制技术使用后能获得的效果已有较多研究报道。有研究观察到使用主动呼吸控制技术后，肺部肿瘤的位移是2.3 mm-4 mm; GTV的动度为0.9 mm-5.9 mm; 位于肺上份的肿瘤位置固定性优于位于肺下份者。有研究^[32]^还发现使用主动呼吸控制技术后，肿瘤在侧方、前后方、上下方的位移分别为（0.3±1.8）mm、（1.2±2.3）mm、（1.1±3.5）mm; 并且当设定的PTV为GTV外放1.5 cm时，使用主动呼吸控制技术可以使肺的受照射体积减少18%。还有研究^[37]^比较了患者深吸气以及30 min后同一呼吸时相使用主动呼吸控制技术的CT图像。2次主动呼吸控制技术扫描的重复性很高，误差在3 mm以内。在1次主动呼吸控制技术过程中膈肌的动度为（1.0±0.9）mm，在2次主动呼吸控制技术过程中为（2.5±1.6）mm。Muralidhar等^[38]^通过iView-GT（iview guided therapy）的DRRs测量了分次内的几何不确定性; 在主动呼吸控制技术使用过程中可以观察到3.2 mm的分次内位移，其系统误差是2 mm-3 mm，随机误差是4 mm-5 mm。Kashani等^[35]^报道了使用CT图像匹配软件测量的主动呼吸控制技术使用过程中肿瘤的平均位移; 吸气和呼气状态下的长时期误差分别是0.3 mm-1.3 mm、0.2 mm-0.7 mm，短时期误差是0.0 mm-0.3 mm。

### 2.3 影像技术的使用

近年来，对于肺部肿瘤放疗而言，最重要的进展就是放疗过程中影像技术的联合，例如CBCT、四维CT（four-dimensional computed tomography, 4D-CT）和PET-CT。影像技术方面的进步、更加精确的剂量计算方法、先进的影像及剂量验证方法的使用保证了高精度放疗的正确实施。

#### 2.3.1 4D-CT

通常也被认为是呼吸运动相关的CT，目前EORTC强烈推荐将其应用于肺部肿瘤放疗计划的制定过程^[15]^。4D是指每次放疗过程中靶区和危险器官的运动。4D放疗最重要的是需要在放疗过程中考虑到呼吸运动的存在，即4D意味着呼吸运动^[15]^。如果放疗过程中不考虑呼吸运动的存在，将会导致随机误差的产生，严重者甚至会导致系统误差。即使对于运动幅度不大的一些原发性肺癌来说，使用4D-CT提供的个体化信息可以也得到更小的PTV边界^[18, 25, 36, 39]^。对于运动幅度较大的肿瘤，其优势会更加突出。

#### 2.3.2 PET-CT扫描

由于PET-CT可以显示比CT扫描更加准确的病变范围，尤其是淋巴结转移的范围，因此已有越来越多的研究结果^[40]^显示PET扫描在放疗计划制定中的优势。患者在PET-CT扫描前的体位固定方法应与CT定位扫描不同，且由于患者在PET-CT扫描过程中的运动会影响图像融合的准确性，所以应在勾画靶区前检查用于融合和匹配的PET-CT图像质量。图像配准时应避免使用非刚性结构，因为尚无针对其在放疗计划中的安全性的评价结果^[41]^。另由于化疗后会出现FDG摄取的快速下降，因此应避免使用化疗后的PET-CT图像进行GTV靶区勾画。

#### 2.3.3 CBCT

肿瘤体积的缩小和机体解剖形态的变化均会导致肿瘤靶区和相应的OARs的分次间变化。而肺癌、前列腺癌、直肠癌、膀胱肿瘤、妇科肿瘤以及头颈部肿瘤等在治疗过程中同样会发生分次内运动。应用同机CT，包括千伏CBCT（kilovoltage cone beam CT, kv-CBCT）、兆伏CBCT（megavoltage cone beam CT, mv-CBCT）或断层治疗，可以获得实时的影像。kv-CBCT由加速器上安装的高能量X射线正交的千伏级X射线管和一个平板式图像探测器组成。mv-CBCT是使用一个安装在机架上的电子射野影像设备（electronic portal imaging device, EPID）的兆伏级的光子线进行成像。断层治疗则是使用一个兆伏级螺旋CT和加速器相结合的放疗设备。将这些同机设备所获得的影像与计划CT的图像进行融合，通过骨性或软组织结构的对比可以得出治疗时患者位置和计划图像的变化大小差值，这个差值可以看作是分次间位移。此外，可以利用放置在患者体表的红外标记对肿瘤进行跟踪，并将其与骨性标志或基准标志物进行配准，可以得到基于人体内部的解剖结构的变化误差大小，从而实现对治疗计划的调整^[8, 42]^。

每种影像技术均有其特点及优势，具体应用时应在详细掌握其特性及应用方法的基础上进行选择。

### 2.4 PTV边界

肺部肿瘤由于存在呼吸动度，因此需要较大的PTV边界。随着4D-CT的逐渐推广，肿瘤的ITV以及呼吸周期中任何一个时间肿瘤的位置均可以确定。后者允许基于肿瘤在一个时间段当中的平均位置进行放疗计划制定和实施治疗^[43]^。当使用门控和跟踪技术时，在外放CTV-PTV边界时还需要考虑到门控窗，并且剂量的计算也需要在适当的呼吸时相进行。因此不能人为对PTV边界进行调整。

### 2.5 剂量的处方和报道

剂量的处方和报道应该遵循国际标准^[9]^。除此之外，对模拟计算的方式和进行计算的CT参数也应该进行报道。以往关于SBRT的研究通常将处方剂量以等中心点剂量的百分比进行报道（如：12 Gy，65%等剂量线）。但现在更倾向于按照剂量体积参数的剂量形式进行处方和报道（如：95%PTV接受12 Gy）。在使用先进的模拟计算方法时，这种剂量的处方和报道方式将使得优化和比较靶体积内部存在高剂量不均匀性的高适形度的放疗技术变得更加容易^[15]^。

### 2.6 射野的安排

原则上，只要剂量分布能够达到临床可接受的标准，所有的技术包括静态、动态和弧形治疗技术均可以应用。但随着治疗时间的延长和非共面野数量的增加，分次内位移会有所增加。因此，EORTC推荐在获得充分的剂量分布的前提下，将治疗时间控制得尽可能短，并尽量使用共面野^[15]^。

### 2.7 剂量体积的控制

很多研究都阐述了剂量体积直方图（dose-volume histogram, DVH）与放疗副反应的相关性。但治疗过程中正常组织会发生一定的变化，因此单纯的放疗前定位CT中的OARs影像并不能确切反映出真实的受照剂量^[44]^。目前越来越倾向于使用一些相关的生物标记物或影像学特点等指标来预测放疗副反应的发生风险，但目前该方法尚处于研究阶段^[45]^。V20（减去PTV后受照剂量≥20 Gy的双肺的百分体积）与肺平均剂量（mean lung dose, MLD）都被认为与放射性肺炎的发生相关^[46]^。尽管V20在35%-37%或MLD在20 Gy-23 Gy之间普遍被认为是安全的，但仍会导致10%-15%的患者在接受低于上述剂量照射时出现严重的放射性肺炎^[47]^。其余的参数，如V5等，其临床意义还需进一步验证。对于SBRT，胸壁以及肋骨也成为OARs。对于胸壁而言，有文献^[48]^推荐其受量应为30 cm^3^受照剂量 < 30 Gy/（3-5）次。对于肋骨而言，当2 cm^3^体积的肋骨受照剂量≤9 Gy/3次时，其骨折发生率约为5%^[49]^。脊髓是串联器官，对全长脊髓而言，常规分割放疗（1.8 Gy/次-2.0 Gy/次）总剂量为45 Gy时，神经元病的发生率 < 1%;总剂量为54 Gy-61 Gy时，神经元病的发生率 < 10%^[50]^。对于SBRT而言，其脊髓最大受照剂量在13 Gy/1次或20 Gy/3次时，神经元病的发生率很低（< 1%）^[15]^。但目前尚缺乏长期观察的数据。

### 2.8 治疗的实施

到目前为止，患者的摆位误差主要通过EPID采集的骨性解剖标志进行验证。但通过kv-CBCT及mv-CBCT获得的影像可以提供肺部肿瘤患者的更为精确的摆位信息^[51]^，并反映分次内位移的信息。对于常规分割的放疗而言，在线调整和离线调整都是适合的。如果预计肺部肿块将在放疗过程中出现较大变化，则需要较为频繁的CBCT扫描（至少每周1次）。如果进行SBRT则需要进行在线调整。当治疗时间较长，如进行SBRT时，甚至还需要在治疗过程中进行重复的CBCT扫描^[51]^。由于CBCT增加了患者的受照剂量，在最终的放疗计划制定时应加以考虑。同样，当使用呼吸控制技术时，如门控或主动呼吸控制技术，则采用CBCT对肿瘤的位置进行验证是非常重要的环节^[52]^。

现代技术的设备如3D-CT和4D-CT、先进的治疗计划系统、EPID、CBCT等越来越多的应用使肺部肿瘤的精确放疗得以实施。但为了确保精确放疗的准确执行，应从患者体位固定、靶区勾画、放疗计划制定和执行等各个环节、步骤加以精确控制和调整。
